# What works for human papillomavirus vaccine introduction in low and middle-income countries?

**DOI:** 10.1016/j.pvr.2017.06.003

**Published:** 2017-06-08

**Authors:** Natasha Howard, Katherine E. Gallagher, Sandra Mounier-Jack, Helen E.D. Burchett, Severin Kabakama, D. Scott LaMontagne, Deborah Watson-Jones

**Affiliations:** a London School of Hygiene and Tropical Medicine, Department of Global Health and Development, Tavistock Place, London WC1H 9SH, United Kingdom; b London School of Hygiene and Tropical Medicine, Clinical Research Department, Keppel St, London WC1E 7HT, United Kingdom; c Mwanza Intervention Trials Unit, National Institute for Medical Research, PO Box 11936, Mwanza, Tanzania; d PATH, Center for Vaccine Innovation and Access, PO Box 900922, Seattle, WA 98109, USA

**Keywords:** Cervical cancer prevention, Human papillomavirus, Vaccination, Low and middle-income countries, Demonstration projects

## Abstract

Since 2007, low and middle-income countries (LMICs) have gained experience delivering HPV vaccines through HPV vaccination pilots, demonstration projects and national programmes. This commentary summarises lessons from HPV vaccination experiences in 45 LMICs and what works for HPV vaccination introduction. Methods included a systematic literature review, unpublished document review, and key informant interviews. Data were extracted from 61 peer-reviewed articles, 11 conference abstracts, 188 technical reports, and 56 interviews, with quantitative data analysed descriptively and qualitative data analysed thematically. Key lessons are described under five themes of preparation, communications, delivery, coverage achievements, and sustainability. Lessons learnt were generally consistent across countries and projects and sufficient lessons have been learnt for countries to deliver HPV vaccine through phased national rollout rather than demonstration projects. However, challenges remain in securing the political will and financial resources necessary to implement successful national programmes.

## 1 Introduction

Cervical cancer, caused by human papillomavirus (HPV), is a leading cause of morbidity and mortality among women in low and middle-income countries (LMICs), with approximately half a million new cases and 266,000 deaths annually [1]. Screening programmes, which have helped reduce mortality rates in high-income countries, are more challenging to establish in low-resource settings [2], [3]. HPV vaccination has emerged as a cost-effective means of preventing over 70% of cervical cancer cases in all resource settings, and the World Health Organization recommends HPV vaccination for girls 9–13 years old [4], [5].

Since 2007, many LMICs have gained experience delivering HPV vaccines through HPV vaccination pilots, demonstration projects and national programmes. Valuable implementation lessons learnt include how to achieve community acceptance, obtain parental consent, and reach adolescent girls for vaccination. Lessons learnt from these country experiences can inform global and national decision-makers how best to implement HPV vaccination, whether through phased introduction or simultaneous national rollout. This commentary summarises major lessons from HPV vaccination experiences in 45 LMICs, which highlight factors that appear crucial for successful HPV vaccination introduction [6], [7], [8].

## 2 Methods

The study involved a systematic literature review, unpublished document review, and key informant interviews [6]. We identified LMICs that had completed at least six months of HPV vaccine delivery through pilot/demonstration projects or national introduction by 30 April 2016. Five peer-reviewed article databases (Medline, Embase, Global Health, Africa-wide Information, ADOLEC) and two unpublished document databases (Open Grey, ProQuest) were searched systematically. Websites of national Ministries of Health, WHO Global Immunisation News, Pan-American Health Organization newsletters, and HPV scientific conference abstracts were searched purposively for unpublished literature and interviewees were asked for national and sub-national technical reports. We conducted semi-structured key informant interviews with purposively sampled technical representatives identified through partners and document searches (e.g. national immunisation programme managers and HPV coordinators).

We extracted document and transcript data to a standardised matrix developed for new vaccine introduction [6], [8]. Topics included national decision-making and planning, service delivery, health workforce, monitoring and evaluation, financial support, sustainability, and scale-up. Qualitative data were analysed thematically, using deductive and inductive coding. Quantitative data (e.g. coverage, adverse events) were analysed descriptively to obtain frequencies, proportions, and scores. The London School of Hygiene & Tropical Medicine Research Ethics Committee provided study approval.

## 3 Results

**Fig. 1:**
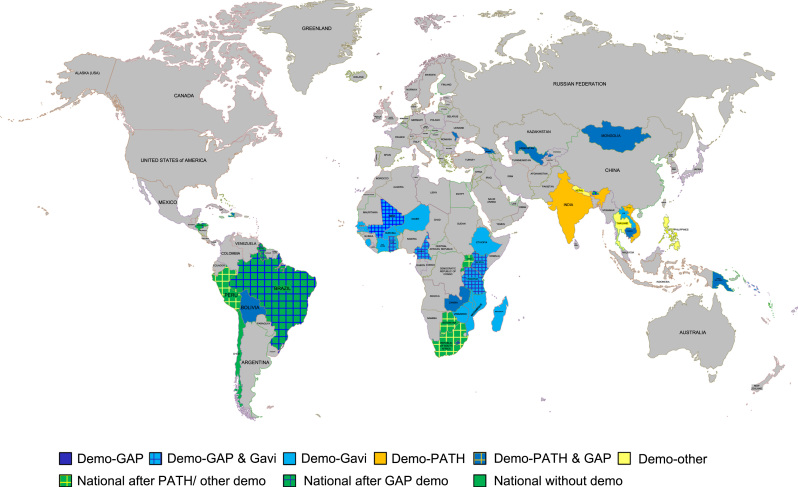
Map of participating countries by project/programme and donor type (as of May 2016). NB: ‘GAP’ is the Gardasil Access Program.

We gathered data from 61 peer-reviewed articles, 11 conference abstracts, 188 technical reports, and 56 key informant interviews (>90% response rate). Forty-six countries were included, as we added one high-income country (Chile) with a novel, one-dose annually, delivery system. Countries provided information from 66 demonstration projects or pilots and 12 national introductions, i.e. 92 distinct HPV delivery experiences (Fig. 1). We present key findings below under five themes of preparation, communications, delivery, coverage achievements, and sustainability (Table 1). Further detailed outputs are available at http://www.rho.org/HPVlessons/ [6], [7], [8].

**Table 1:** Themes and findings on what works for HPV vaccination.

Preparation	Ensure high-level political commitment.
	Encourage inter-ministerial collaboration early, particularly for health, education, and finance.
	Allow sufficient time for planning and micro-planning.
Communication	Allow enough time for social mobilisation.
	Use clear messaging, focusing on cancer prevention and how to be vaccinated.
	Include face-to-face communication with credible influencers.
	Respond quickly and thoroughly to rumours and negative media.
	Use consent procedures that are consistent with routine immunisation.
Delivery	Ensure availability of accurate population data or time and funds for enumeration.
	Multiple delivery strategies could be useful within the same country.
	Implement a two-dose rather than a three-dose HPV vaccination schedule if possible.
	Use routine delivery approaches.
	Use community health-workers to help identify missing and out-of-school girls.
Coverage	Implement a two-dose vaccination schedule for higher completion rates.
	Use school-based delivery to obtain high coverage.
	If using grade-based delivery, consider including age in reporting forms.
Sustainability	Ensure sufficient time and resources to calculate accurate costing estimates for national rollout of HPV vaccine and delivery.
	Choose a delivery strategy that is feasible for national scale-up.
	Advocate for sufficient funding to achieve successful national scale-up.

### 3.1 Preparation

Three key preparation lessons emerged. First, high-level political commitment contributed to project and national programme effectiveness, e.g. by increasing HPV vaccine prioritisation and interest, galvanising collaboration between partners, and strengthening commitments to financing and delivery. Second, early inter-ministerial collaboration was crucial. Collaboration between health and education ministries enabled cooperation between teaching and healthcare staff. Engaging private schools early in the planning process encouraged their participation. Collaboration between health and finance ministries helped ensure timely funds release. Third, the new target population and delivery strategies required substantial microplanning and development of new collaborations between institutions that may not have worked together previously. Insufficient microplanning led to considerable problems, particularly for school-based delivery, which was often new, and where target population numbers needed enumeration.

### 3.2 Communication

Five key communication lessons emerged [7]. First, effective community mobilisation activities required implementation at least one month prior to vaccination and used multiple channels. Second, the most effective messages emphasised cancer prevention, vaccine safety, and national and global endorsement (e.g. HPV vaccination prevents cervical cancer, is safe, will not harm future fertility, and is endorsed by the government and the World Health Organization), while explaining clearly where and when girls could be vaccinated. Third, face-to-face communication between credible influencers (e.g. teachers, health-workers, community leaders), parents, and communities enhanced support and mitigated rumours. Fourth, rumours and negative publicity were best addressed quickly and comprehensively, e.g. using several communication channels (celebrity champions, WHO and government endorsement). Fifth, successful consent procedures were consistent with those used for routine immunisation. While opt-out consent was easier logistically, opt-in consent could generate misunderstanding and mistrust in communities.

### 3.3 Delivery

During the period analysed, over 1.7 million girls were reached and 1.4 million were fully vaccinated [6]. While many delivery lessons were similar to those for routine vaccination, aspects of HPV vaccination were new (e.g. target population, usage of schools). Five key delivery lessons emerged. First, enumerating the population of potentially eligible girls before vaccination proved challenging and expensive but necessary because existing population data were normally unreliable or inaccurate. Investing in enumeration the first year improved preparation in future years, particularly in terms of vaccine register development and stock planning. Time and effort required to enumerate and then track girls between doses was often underestimated. Second, different delivery strategy mixes (e.g. schools, health facilities, outreach) could work in different contexts within the same country, as logistics and school enrolment were not homogeneous across each country. Third, implementing a two-dose vaccination schedule was easier and cheaper than a three-dose schedule, as the period analysed included the initial shift from three-dose to two-dose schedules. Delivery of all doses within one school year minimised dropout and improved coverage, while providing a second vaccination opportunity successfully reached girls and parents who initially refused or were absent/out-of-school. Fourth, using routine vaccination programme infrastructure and resources (e.g. transport, cold chain, staff) was easier and more efficient than separate HPV-specific transport, storage, or delivery. Fifth, mobilising community health-workers (CHWs) to assist in identifying out-of-school girls and those who missed doses improved coverage. Enumerating and vaccinating out-of-school girls was particularly difficult, with no adequate strategies identified other than CHWs mobilising them to self-present at health facilities.

### 3.4 Coverage

All 51 (77%) demonstration projects and 9 (75%) national programmes with complete data achieved more than 50% final-dose coverage, with 50 (83%) achieving 70% or higher coverage. Three key coverage lessons, related to those for delivery, emerged. First, dropout rates between doses appeared somewhat lower in countries implementing a two-dose vaccination schedule, though data were insufficient to confirm this. Coverage levels, rivalling some high-income country programmes, indicate high vaccine acceptability and possible contributions of dedicated investment and – for Gavi demonstration projects - emphasis on achieving high coverage in the first years. Coverage thus requires further monitoring so that if coverage rates drop as HPV vaccination becomes routine or delivery strategies change, remedial measures can be organised. Second, school-based delivery strategies were feasible and attained high coverage, though initial efforts were necessary to coordinate with the Ministry of Education and school leadership. Third, grade-based eligibility was logistically easier to implement than age-based in schools, but resulting data were more challenging to reconcile with health information systems that were organised around age-based data. Thus, age-based eligibility was easier for estimating coverage and uptake rates.

### 3.5 Sustainability

Estimated recurrent financial delivery costs, excluding vaccines, ranged from US$1.11 to 9.21 per dose and differed by funding source. Three key financial sustainability lessons emerged. First, accurate costing of both vaccines and delivery were critical for countries to estimate the financial resources needed for programme sustainability. Second, strategies that worked well in demonstration projects were not always feasible for national scale-up, e.g. due to costs or lack of staff. Third, funding uncertainties negatively influenced country decisions to scale-up HPV vaccination nationally. High HPV vaccine costs combined with high perceived delivery costs that countries must co-finance in future, appeared to discourage many policymakers and budget-holders from committing to HPV vaccination or to change to lower-cost untested delivery approaches (e.g. facility-based delivery). Thus, rigorous impact data (e.g. economic, human resources) and ongoing technical assistance with budget planning and economic analyses can further support the design of sustainable national HPV vaccination strategies.

## 4 Conclusions

Lessons learnt were generally consistent across demonstration projects and supported by smaller studies [9]. However, small project size, district selection processes, and the desire to demonstrate high coverage quickly, made some lessons inapplicable to national rollout. In future, phased national rollout may provide the benefits of demonstration projects with the added advantage of maintaining political commitment to scale-up. Sufficient lessons have been learnt for countries to deliver HPV vaccine through phased national rollout or national programmes rather than demonstration projects. Countries now have empirical evidence on the factors that lead to successful HPV vaccination, yet challenges remain for some countries in securing the political will and financial resources necessary from governments, donors, and partners to implement successful national programmes. This is the next major challenge to ensuring potential HPV vaccine benefits in reducing cervical cancer morbidity and mortality are achieved in countries with the highest burden.

## Conflict of interest

None declared.
